# Nasal Polypus Cured with Sanguinaria Canadensis

**Published:** 1840-09

**Authors:** 


### NASAL POLYPUS CURED WITH SANGUINARIA CANADENSIS.

Being lately in Newark, Ohio, Dr. Brice, for more than 30 years a respectable practitioner of that place, narrated to us three cases of polypus of the nostril, which he had permanently cured by the application of the root of the sanguinaria canadensis. One of the patients was a youth, in whom the polypus projected out of the nostril. A physician in a neighboring town tore away a part or the whole of it, and the operation was followed by profuse hemorrhage. Sometime afterwards the doctor saw him, and the polypus again extended beyond the alœ nasi. The application of the powdered root and the decoction of the sanguinaria soon caused it to assume a pale color and shrink up. Under the continued use of the medicine he entirely recovered.

Another patient was a little girl, in whom the polypus was distinctly seen, but it did not present itself entirely. The same applications effected a radical cure.

A third was a man rather advanced in life, whose nose was much obstructed by the size of the polypus, but it did not descend to the lip. It was permantly removed by the same treatment.

We do not recollect to what extent the sanguinaria has been employed in the treatment of polypus, and are writing these memoranda remote from all books of reference. Should the reader be already familiar with the use of this remedy, he cannot charge us with prolixity in this testimony of its efficacy. D.

